# Editorial: Atopic march and atopic multimorbidity

**DOI:** 10.3389/falgy.2026.1872350

**Published:** 2026-05-22

**Authors:** Lizette M. Cortes, Evan S. Dellon

**Affiliations:** 1 Department of Medicine, College of Medicine, Duke University, Durham, NC, United States; 2 Department of Population Health and Pathobiology, College of Veterinary Medicine, North Carolina State University, Raleigh, NC, United States; 3 Center for Esophageal Diseases and Swallowing, and Center for Gastrointestinal Biology and Disease, Division of Gastroenterology and Hepatology, University of North Carolina School of Medicine, Chapel Hill, NC, United States

**Keywords:** atopic march, atopic multimorbidity, barrier disfunction, demodex, mast cells, mitochondria, textile dyes

The incidence of allergic diseases has increased dramatically over recent decades (1), accompanied by growing recognition of multimorbidity among atopic conditions, including atopic dermatitis (AD), food allergy, allergic rhinitis, asthma, and eosinophilic esophagitis (EoE) (2). These disorders frequently occur sequentially over time in what has been termed the atopic march (3), suggesting shared upstream mechanisms driving immune dysregulation across epithelial tissues (4). Although genetic susceptibility contributes to disease risk (5), the rapid rise in allergic disease strongly implicates environmental factors that disrupt epithelial barriers and alter immune homeostasis (6). This editorial will summarize new concepts discussed and published in the research topic “Atopic March and Atopic Multimorbidity”.

A central role for barrier integrity in early disease initiation is highlighted by the prevention study described by Inuzuka et al., who evaluated moisturizer application in high-risk neonates. This randomized, controlled protocol builds on prior observations suggesting that early enhancement of skin barrier function may reduce the development of AD. The hypothesis that frequent application of moisturizer in neonates can prevent AD underscores the importance of epidermal barrier integrity as a critical early determinant of allergic disease. These findings support the concept that barrier disruption precedes immune sensitization and that restoring epithelial integrity may alter the trajectory of the atopic march (7).

While genetic factors such as filaggrin deficiency contribute to barrier dysfunction, environmental exposures likely represent additional triggers (8). Experimental work by Cortes et al. investigating disperse textile dyes provides evidence that common environmental chemicals can directly impair epithelial function. Exposure of keratinocytes and intestinal epithelial cells to disperse dyes reduced cell viability and impaired mitochondrial respiration. These findings were accompanied by alterations in inflammatory signaling and genes associated with barrier integrity. In complementary studies, Cortes et al. further demonstrated that specific dyes induced pro-inflammatory cytokine responses in peripheral blood mononuclear cells and disrupted epithelial tight junction-related pathways. Notably, these effects were dependent on chemical structure, suggesting that physicochemical properties influence epithelial penetration and toxicity.

Mitochondrial dysfunction represents a compelling mechanism linking environmental exposure to allergic disease. Mitochondria regulate epithelial barrier maintenance, oxidative stress responses, and inflammatory signaling (9). Chemical-induced impairment of mitochondrial respiration can alter tight junction integrity, modify lipid metabolism, and promote release of danger-associated molecular patterns (10). These events may activate innate immune pathways and promote type 2 inflammation. Thus, environmentally induced mitochondrial stress may represent an upstream trigger in the atopic march (11).

In parallel, biological factors within the follicular ecosystem may amplify immune activation once barrier integrity is compromised. In a hypothesis-raising article, Senior-Fletcher proposed that Demodex mites may contribute to allergic disease progression. Reduction of Demodex reproduction using petroleum jelly was suggested to be associated with improvement not only in blepharitis and dermatitis but also in rhinitis, asthma, and other allergic symptoms. Although these observations are preliminary and require formal validation, they raise the possibility that mite-associated antigens, microbial components, and inflammatory debris may contribute to chronic immune stimulation.

Within the context of epithelial barrier disruption, Demodex may function as ecological responders to environmental change (12). Chemical exposures such as textile dyes may alter epithelial metabolism, disrupt lipid composition, and modify microbial communities. These changes could influence the follicular microenvironment and potentially affect Demodex density or antigen exposure. Mite-derived proteins, chitin-like components, and associated bacteria may then activate epithelial alarmins and innate immune signaling pathways. This cascade could promote type 2 immune polarization and systemic allergic sensitization (12).

This model suggests a chemical-barrier-follicular axis in the initiation of the atopic march. Environmental chemicals may impair mitochondrial function and epithelial integrity (11). Barrier disruption may then alter follicular ecology, including microbial communities and Demodex. These changes, in turn, may amplify local immune activation and promote systemic type 2 inflammation (13). Over time, this process may contribute to progression from cutaneous disease to gastrointestinal and respiratory manifestations.

Support for the importance of barrier integrity in allergic sensitization is further reinforced by the porcine peanut allergy model described by Shakya et al., in which transcutaneous sensitization following barrier disruption led to systemic allergic responses. These findings emphasize that the skin functions as a critical immunological interface capable of initiating systemic disease.

Mast cells may represent a central integrator within this framework (14). Both chemical-induced epithelial stress and mite-associated inflammation can activate mast cells, which release mediators that increase vascular permeability, recruit eosinophils, and amplify type 2 inflammation (14). This suggests that mast cells may link environmental exposures, epithelial damage, and follicular biology in the progression of the atopic march.

Together, these studies support a unifying hypothesis: environmental chemical exposures that impair mitochondrial function and epithelial integrity may initiate allergic disease, while follicular organisms such as Demodex may amplify and sustain immune activation. This integrated model highlights the importance of considering the skin as a dynamic ecological system influenced by chemical, microbial, and host factors.

Future studies should investigate the impact of environmental chemicals on epithelial mitochondrial function, barrier integrity, and follicular ecosystems combined. In addition, the potential role of Demodex and associated microbiota in modulating allergic inflammation warrants further exploration. Translational models that recapitulate epithelial sensitization and systemic allergic progression will be particularly valuable in testing these hypotheses. The porcine model has been successfully used to study atopic diseases such as EoE (15), and the role of local mast cells in a porcine model of eosinophilic esophagitis has been reported (16).

Understanding the interactions between environmental exposures, epithelial metabolism, and follicular ecology may provide new insights into the origins of the atopic march. Identification of upstream triggers could enable preventive strategies targeting barrier integrity and environmental exposure, ultimately reducing the burden of allergic disease.
